# Community Participation and Subjective Wellbeing: Mediating Roles of Basic Psychological Needs Among Chinese Retirees

**DOI:** 10.3389/fpsyg.2021.743897

**Published:** 2021-10-22

**Authors:** Lanshuang Chen, Zhen Zhang

**Affiliations:** ^1^Key Laboratory of Behavioral Sciences, Institute of Psychology, Chinese Academy of Sciences, Beijing, China; ^2^Department of Psychology, University of Chinese Academy of Sciences, Beijing, China

**Keywords:** community participation, subjective wellbeing, basic psychological needs, retirees, mediating role

## Abstract

**Objectives:** Participation in various social organizations, including community organizations, has become an important part of later year. The current study examined the effects of community participation on subjective wellbeing (SWB) and mediating mechanisms among retired residents. Specifically, this study attempts to explain the link between community participation and SWB from the perspective of basic psychological needs (BPN).

**Methods:** A total of 1,458 community-dwelling retirees aged ≥50years in China participated in this study. A self-developed questionnaire measured the general levels of community participation. BPN were assessed with the BPN Scale. SWB was represented by life satisfaction, positive affect, and negative affect.

**Results:** Community participation positively predicted one’s SWB. Specifically, retirees with a higher levels of community participation often indicated higher life satisfaction and positive affect, and lower negative affect. Three BPN, which consist of needs for autonomy, competence, and relatedness, mediated the effect of community participation on SWB, respectively, after controlling for some main demographic and socioeconomic factors.

**Conclusion:** The BPN perspective provides a comprehensive explanation for understanding the link between community participation and SWB. Policymakers should consider the role of community participation when developing measures to improve retirees’ quality of life.

## Introduction

Participation, health, and security are regarded as the three core elements of active aging (United Nations, 2002). While retirees withdraw from job roles, they have more time to do what they are interested in Shultz and Wang (2011) and Moen and Flood (2013). China is among the fastest aging countries in the world. The size of retired population is quickly expanding and had exceeded 123 million by the end of 2019 (National Bureau of Statistics of China, 2020). Here, community participation has emerged as a crucial way of remaining socially active after retirement (Tong, 2016; Zhang, 2019). Such community-based organizational participation plays a significant role in providing support and informal care, in addition to services provided by family and governments (United Nations, 2002). Considerable research has confirmed the benefits of community participation for one’s subjective wellbeing (SWB; Harlow and Cantor, 1996; Zhang and Zhang, 2015; Li et al., 2018). Compared with non-retired or younger adults, retirees or older adults are often more dependent on services and resources of residential community or neighborhood. The interaction between neighbors by community participation can greatly enrich the later life of retirees and strengthen neighborhood ties, which are closely related to their physical and mental health. Nevertheless, empirical research on the mechanisms of this relationship remains insufficient. Furthermore, although China has the largest number of retirees globally, relatively few studies have examined community participation and its psychological outcomes among retirees of China. The current study aimed to investigate the community participation of Chinese retirees, its effects on SWB, and the related mechanisms, which is expected to provide some references for government managers and policy makers on community governance and coping population aging.

### Community Participation

Concept of community participation is relatively ambiguous and context-dependent. Narrowly speaking, it refers to the degree of one’s active engagement in various organizations or groups that affect their lives and the community itself, as a member within the residential community (Ohmer, 2007; Talò, 2018). Community participation can be understood through several aspects. First, it should be relevant to the local residential community of retirees. Second, it should reflect sociality of participation and often be conducted in a form of organization or group. Finally, it should be a comprehensive concept, covering main aspects of local community activities or affairs. In China, community participation is more geared toward resolving community difficulties or neighborhood conflicts, offering informal services for community residents, organizing or participating in cultural and recreational activities, and so on. For example, in urban China, “neighborhood committee aunt” (居委会大妈) has become an informal title for middle- and old-aged women who engage in community governance and services.

It is well established that community participation influences physical and psychological health-related outcomes among the elderly, including better self-reported physical functioning (Sirven and Debrand, 2008; Gilmour, 2012), improved wellbeing (Betts et al., 2011), decreased loneliness (Rogers et al., 2021), and lower depressive symptoms (Glass et al., 2006; Park et al., 2013).

Despite a rich literature on the link between social participation and SWB, few studies have focused on the role of social participation in SWB in the context of residential community. Moreover, several common theories on the effect of participation on SWB often provide corresponding explanations from a single perspective. The activity theory of aging contends that older adults who adjust to later life changes by remaining active in social participation are healthier and happier (Lemon et al., 1972; Lampinen et al., 2006). Activities offer opportunities for individuals to enhance their sense of personal control and self-worth through achieving goals, thus promoting high quality of life among older adults (Dai et al., 2013). According to the social capital theory, having access to informal organizations partly offsets the negative effects of shrinking social networks associated with retirement (Swaroop and Morenoff, 2006). Community participation can expand the size and diversity of one’s social network and reflect one’s adjustment to a new way of life after retirement. Furthermore, community organizations provide channels for receiving various supportive resources (Putnam, 2000; Lin, 2001). Role theory emphasizes that community participation helps retired people to acquire a new identity by autonomously engaging with various civic organizations (Evandrou and Glaser, 2004). Although the above theories provide some explanations for the link between participation and SWB, a more comprehensive theory that integrates these concepts remains necessary.

### The Roles of BPN

The basic psychological needs (BPN) perspective as a macro theoretical approach, which integrates activity theory, social capital theory, and role theory to some extent, can further clarify how community participation affects retirees’ SWB. We infer that community participation can influence retirees’ SWB by multiple psychological needs. This concept to some extent integrates the interpretation mechanisms of the above theories.

BPN is a sub-theory of self-determination theory (SDT) proposed by Ryan and Deci (2017). This theory proposes that from an organismic perspective, humans have evolved intrinsic tendencies for survival, development, and flourishing. These intrinsic tendencies can be summarized as needs for competence, autonomy, and relatedness. The BPN is fundamental for one’s development, wellness, and vitality, while frustration of BPN can cause adverse health effects (Ferrand and Martinent, 2020). The need for autonomy describes the inclination of individuals to experience self-endorsement and ownership of their actions. That is, individuals tend to control and regulate their actions by their own will. The need for competence refers to having a sense of self-efficacy in one’s interactions with the social environment. That is, individuals can develop and experience a feeling of competence or achievement by participating in various tasks and accomplishing a specific aim. The need for relatedness contends that people need to feel connected and experience a sense of belongingness or importance to others in their social group, while avoiding rejection and disconnectedness. That is, people who have deep and meaningful relationships with others tend to be important to themselves. According to Ryan and Deci (2017), the three basic needs are both relatively independent and highly intercorrelated. On the one hand, they have unique effects on wellbeing. For instance, Neubauer et al. (2017) suggested that competence rather than autonomy was associated with SWB of old adults aged over 87. For nursing home residents, relatedness was more important than competence and autonomy in the caring relationship (Custers et al., 2012). On the other hand, they are mutually implicated and complementary. For example, one can experience a feeling of competence only when feeling autonomy in an activity. Moreover, the needs for autonomy and relatedness considered as essential components of optimal functioning are positively related to SWB (Ferrand et al., 2014).

The link between BNP and SWB has been confirmed across diverse cultures (Church et al., 2013) and all age groups, including the elderly (Blazer et al., 2007; Custers et al., 2010; Neubauer et al., 2017; Kloos et al., 2019; Tang et al., 2020). Through a systematic review and meta-analysis, Tang et al. (2020) reported a positive correlation between BPN and various positive indicators of wellbeing. They also revealed a negative correlation between BPN and negative indicators of wellbeing, such as depression and apathy. Some studies involving older adults have suggested mediating roles of BPN (Gagné, 2003; Kanning and Schlicht, 2008; Martin et al., 2019; van der Kaap-Deeder et al., 2021). Kanning and Schlicht (2008) proposed that the effect of physical activity on SWB depended on needs for autonomy, competence, and relatedness. A new study suggested that BPN largely explained the relationships between ego integrity and life satisfaction among people with an age over 65years (van der Kaap-Deeder et al., 2021).

Generally, community participation could be thought to enhance one’s BPN, especially among older adults who have retired from work and some social roles as it is not particular investigated yet. High-frequency participation expands one’s social network and enhances one’s sense of relatedness. According to the social benefit hypothesis (Li and Ferraro, 2005), community participation influences SWB by helping retirees increase supportive social relationships. In a new social group or organization, an individual can build novel connections and friendships, thus meeting one’s need to care for and be cared for by others. High-frequency participation in various activities also helps retired people enhance their feelings of autonomy. Unlike completing obligatory work tasks before retirement, community participation after retirement is often a voluntary, self-determined action. In addition, positive participation enhances one’s self-efficacy in a specific field. Based on the above literature and theories, community participation and SWB are closely related, and community participation may influence SWB through BPN.

**Figure 1:**
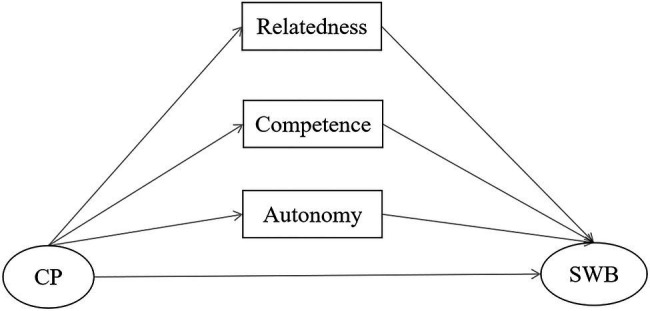
Research framework. CP and SWP represent community participation and subjective wellbeing, respectively.

Thus, we sought to determine whether the three BPN mediate the relationship between community participation and SWB. Considering the comprehensiveness of the BPN concept, we suggest that it can likely explain the relationship between community participation and SWB (see research framework in Figure 1).

### Current Study and Hypotheses

Using community-dwelling retirees as participants, this study attempted to confirm the positive role of community participation in SWB, as well as the potential mediating roles of the BPN for competence, relatedness, and autonomy. The hypotheses are as follows:

Hypothesis 1: Community participation is positively associated with SWB indicated by life satisfaction, positive affect, and negative affect.

Hypothesis 2: The three psychological needs are positively associated with SWB indicated by life satisfaction, positive affect, and negative affect.

Hypothesis 3: Community participation is positively associated with the three psychological needs.

Hypothesis 4: Three BPN mediate the correlation between community participation and SWB.

## Materials and Methods

### Participants and Design

The ethics committee of the corresponding author’s affiliation approved this study. In China, the official retirement age is 50 or 55years for women, 60years for men. We thus recruited community-dwelling retirees aged 50years or older as participants from urban communities in four cities (Nanjing, Jinling, Shijiazhuang, and Cangzhou) across Hebei, Shandong, and Jiangsu provinces. The investigation was conducted by project directors and psychology graduate students, with the assistance of cadres of local communities. The questionnaire was linked to “Star of Questionnaire,” an online platform for professional questionnaire surveys. Community workers forwarded the link of the online survey to the mobile phone of every recruited participant. Participants completed and submitted the survey by mobile phone, independently and anonymously. Before answering questionnaires, participants read and offered informed consent statement by mobile phone. Of the 1700 questionnaires distributed, 1,458 were returned and valid (valid response rate=85.8%). Respondents included 626 men and 832 women. Characteristics of the participants in terms of background variables could be found in Table 1. Each participant took approximately 20min to complete and submit the survey. Finally, each participant was compensated with RMB 15 (about USD 2) *via* WeChat-Pay, the predominantly used online payment platform in China.

**Table 1:** Demographic and socioeconomic characteristics of participants (*N*=1,458).

Variable	n	valid %
**Gender**		
Male	626	42.94
Female	832	57.06
**Age**		
50–54years	135	9.26
55–59years	482	33.06
60–64years	368	25.24
65–69years	303	20.78
70–74years	101	6.93
75–79years	53	3.64
80 and older	16	1.10
**Educational level**		
Elementary or lower	150	10.29
Junior high school	459	31.48
Senior high school	604	41.43
Junior college or higher	245	16.80
**Marital status**		
Without a spouse	140	9.60
With a spouse	1,318	90.40
**Monthly income (RMB)**		
Less than 500	42	2.88
500–1,000	22	1.51
1,000–1,500	38	2.61
1,500–2000	91	6.24
2000–2,500	143	9.81
2,500–3,000	219	15.02
3,000–4,000	276	18.93
4,000–5,000	222	15.23
5,000–6,000	139	9.53
More than 6,000	266	18.24
**Physical condition**		
Very poor	11	0.75
Poor	42	2.88
Fair	523	35.87
Good	647	44.38
Excellent	235	16.12

### Measurements

#### Community Participation Survey

The extant literature lacks a universally accepted measurement of community participation. As a nationally representative database, the China Health and Retirement Longitudinal Study (CHARLS) investigated the social or group participation of residents aged 45years and older. It examined the following aspects: friends and family gatherings, community residents’ organizations, sports or recreational organizations, and voluntary or charity work organizations, while recent research shows that community participation usually takes the following forms: recreational and sports groups, religious organizations, volunteer organizations, and political or civic engagement organizations (Albanesi et al., 2007; Cicognani et al., 2008).

Referring to the extant instruments and empirical research on community participation, and social and cultural background of China, five types of organizations were selected as primary forms of community participation. These organizations are often informal and focus on community affairs, services, hobbies, and other activities. They are as: (1) community residents’ organizations or neighborhood committees that assist in managing community affairs, reconcile conflicts or disputes between neighborhood residents, bridge the communication between residents and local governments or management agencies, and so on; (2) volunteer service associations that organize various public benefit activities and assist in maintaining the security, cleaning, and traffic management in residential communities; (3) wedding and funeral councils or similar organizations of residential communities, which help organize, conduct, and supervise wedding and funeral ceremonies, which is an important form of mutual help between neighbors in China; (4) organizations based on interests, hobbies, and training, such as art, music, or educational organizations; and (5) community charitable organizations intend to help people who are poor or having difficulties by organizing donations and offering support or services. Volunteer organizations are common community organizations in both Chinese and Western culture, while residents’ committees and wedding and funeral councils as community organizations are unique to China. Participants were asked to rate their level of participation in the above-mentioned organizations using a five-point Likert-type scale (from *1=never* to *5=always*).

Cronbach’s alpha coefficient for all items was.79, and coefficients of all pairwise correlations were positive and significant. KMO (0.83) and Bartlett’s test (*χ*^2^=2095.60, df=10, *p*<0.001) indicated that the scale was acceptable for factor analysis. Exploratory factor analysis showed that only one eigenvalue was above 1.0 (2.79). This single factor accounted for 55.9% of the total variance, and the factor loadings of all items were between 0.60~0.80.

Several indices were employed to evaluate the goodness-of-fit of path models, including χ^2^/*df*, Comparative Fit Index (CFI), Tucker-Lewis Index (TLI), Root Mean Square Error of Approximation (RMSEA), and Standardized Root Mean Square Residual (SRMR). Model fit was acceptable when the TLI values were 0.90 or above, CFI values were 0.90 or above, SRMR values were 0.08 or below, and RMSEA values were 0.08 or below (Brown, 2015). Confirmatory factor analysis showed that the model had an acceptable goodness-of-fit (*χ*^2^=37.49, *χ*^2^/df (5)=7.50, CFI=0.98, TLI=0.97, RMSEA=0.07 [90% CI:0.05–0.09], SRMR=0.02). Thus, community participation survey as a self-developed and validated questionnaire can reflect the degree of participation of residents in community activities, and the general mean score as an indicator of the degree of community participation was acceptable.

#### Basic Psychological Needs Scale

Original scale developed by Gagné (2003) aims to assess one’ levels of needs for autonomy, competence, and relatedness. The higher scores indicate more satisfaction with BPN. Reliability and validity of Chinese version of Basic Psychological Needs Scale (BPNS) have been confirmed (Liu et al., 2013; Wei et al., 2020). The Chinese version of BPNS contains 6 items for autonomy (e.g., “*I feel like I can decide for myself how to live my life*”), 6 items for competence (e.g., “*I often do not feel capable*”), and 7 items for relatedness (e.g., “*I really like the people I interact with*”). The items are rated on a 5-point Likert scale, ranging from 1 (*Completely Disagree*) to 5 (*Completely Agree*). The Cronbach’s alpha coefficients for autonomy, relatedness, and competence subscale were, 0.74, 0.79, and 0.72, respectively, in the current sample.

According to Diener et al. (1999), SWB comprises three core components: high life satisfaction, high positive affect, and low negative affect. Thus, in the current study, SWB was indicated by the above three indicators.

#### The Satisfaction With Life Scale (SWLS)

The SWLS is one of the most popular measures used to assess an individual’s general sense of fulfillment in life (Diener et al., 1985; Zhang and Zhang, 2017; Fastame et al., 2021). It comprises five items, and each item is rated on a 5-point Likert-type scale (from *1=strongly disagree* to *5=strongly agree*). Sample items include, “*The conditions of my life are excellent*.” The reliability and validity of the Chinese version have been verified (Bai et al., 2011). In the current study, Cronbach’s alpha coefficient was. 89.

#### Positive Affect and Negative Affect Schedule (PANAS)

The PANAS has two dimensions that describe one’s positive and negative emotions, respectively (Watson et al., 1988). On a five-point scale, participants rate the extent of their feelings of positive affect (PA; e.g., *excited*) and negative affect (NA; e.g., *upset*) during the past month. The Chinese version is confirmed to be reliable and valid (Liang and Zhu, 2015). In the current study, a short version of the PANAS with 12 items was used, and each subscale consisted of 6 items (Kercher, 1992). The Cronbach’s alpha coefficient was.88 for the PA and.86 for the NA, respectively, in the present study.

#### Covariates

In the current study, we assessed several demographic and socioeconomic variables. These included sex, age (from “*1=50~54years*” to “*7=more than 80years*”), marital status (with a spouse and without a spouse [separated/divorced/widowed/never married]), education level (from *1=elementary or lower* to *4=junior college or higher*), self-reported monthly income (from *1=less than 500 RMB* to *10=more than 6,000 RMB*), and physical health. Physical health was assessed by the following question: “Compared to people your own age, please rate your overall physical health on a five-point scale (from *1=very poor* to *5=Excellent*).” The grade scores of self-reported income and physical health were used as continuous variables in the analysis. Researchers suggest that these factors are associated with SWB (Pinquart and Sörensen, 2000; Morrow-Howell, 2010). Health and financial status are often identified as two of the most important determinants of SWB (Pinquart and Sörensen, 2000; Zhang and Zhang, 2015). Good physical condition and economic condition can provide retirees with more opportunities or possibilities to participate into various community organizations, and then enhance retirees’ SWB (Pinquart and Sörensen, 2000).

### Statistical Analysis

SPSS 22.0 was used for descriptive statistics and correlation analyses. M*plus* 8.0 (Muthén and Muthén, 2017) was adopted to test the mediation model and all direct and indirect effects. Community participation with the five items was hypothesized as the latent predictive variable. The three BPN were hypothesized as independent mediating variables. SWB served as a latent outcome variable indicated by life satisfaction, negative affect, and positive affect. Finally, the effects of demographic and socioeconomic factors on mediating variables and dependent variables were controlled in the mediation analysis processes. Several indices were employed to evaluate the goodness-of-fit and structural models, including *χ*^2^/*df*, CFI, TLI, RMSEA, and SRMR.

## Results

### Multicollinearity Diagnosis

Given that there are possible linear correlations among three BPN, variance inflation factor (VIF) is used to evaluate multicollinearity. When VIF is less than 10, multicollinearity between variables can be ignored (Schroeder et al., 1990). The results showed that the VIF indexes of the needs for relatedness, competence, and autonomy were 2.209, 2.306, and 2.209, respectively, which indicated that the effect of multicollinearity was small and could be ignored.

### Descriptive Findings

Preliminary analyses revealed the level of community participation according to five aspects among Chinese retirees. For example, approximately 7.6% of retirees participated in community residents’ organizations at a high level (from 4=*often* to 5=*always*); 24.2% in volunteer service associations; 12.4% in charitable organizations; 13.5% in wedding and funeral councils; and 21.5% in organizations based on interests, hobbies, and training. Further analysis indicated that approximately 40% of retirees reported a relatively high level of participation in least one community organization (selecting 4 *=often* or 5 *=always* on a five-point scale; see Table 2).

**Table 2:** Basic information of community participation (*N*=1,458).

	M(*SD*)	Never	Occasionally	Generally	Often	Always
1 Community residents’ organizations	1.88(0.90)	529(36.28%)	707(48.49%)	111(7.61%)	89(6.10%)	22(1.51%)
2 Charitable organizations	2.42(0.89)	128(8.78%)	818(56.10%)	331(22.70%)	134(9.19%)	47(3.22%)
3 Wedding and funeral councils	2.24(1.05)	352(24.14%)	661(45.34%)	248(17.01%)	138(9.47%)	59(4.05%)
4 Organizations based on interests, hobbies, or education	2.44(1.21)	365(25.03%)	505(34.64%)	275(18.86%)	208(14.27%)	105(7.20%)
5 Volunteer service associations	2.73(1.09)	121(8.30%)	627(43.00%)	357(24.49%)	230(15.78%)	123(8.44%)

### Correlation Analyses

The correlations between community participation and three indicators of SWB were moderate and significant. Community participation was significantly associated with need for autonomy, relatedness, and competence. The three BPN were also significantly associated with all three indicators of SWB. These findings indicate that community participation may be an important predictor of three psychological needs and various indicators of SWB (see Table 3). Simultaneously, the research results also showed that there were varying correlation between the covariates and the main variables. Especially, physical condition and monthly income were significantly correlated with all the major variables, which meant that the controlling the roles of covariates was necessary.

**Table 3:** Inter-correlations between main variables (*N*=1,458).

	M(*SD*)	1	2	3	4	5	6	7	8	9	10	11	12
1 Age
2 Sex		−0.33^***^											
3 Education		0.05	−0.14^***^										
4 Material status		−0.08^**^	−0.11^***^	0.06^*^									
5 Monthly income		0.19^***^	−0.22^***^	0.49^***^	0.08^**^								
6 Physical condition		−0.04	0.05	0.13^***^	0.05^*^	0.11^***^							
7 Community participation	2.34(0.76)	−0.08^**^	0.03	−0.12^***^	0.04	−0.14^***^	0.20^***^						
8 Relatedness	4.05(0.59)	0.05	0.15^***^	0.06^*^	0.04	0.06^*^	0.30^***^	0.28^***^					
9 Competence	3.86(0.59)	0.00	0.05^*^	0.19^***^	0.07^**^	0.15^***^	0.36^**^	0.22^***^	0.68^***^				
10 Autonomy	3.74(0.57)	0.06^*^	0.08^**^	0.10^***^	0.06^*^	0.13^***^	0.32^**^	0.18^***^	0.69^***^	0.70^***^			
11 Life satisfaction	3.84(0.81)	0.07^**^	0.07^*^	0.02	0.11^**^	0.11^***^	0.37^**^	0.25^***^	0.56^***^	0.48^***^	0.56^***^		
12 Positive emotions	3.49(0.81)	−0.02	0.00	0.12^***^	0.06^*^	0.10^***^	0.44^**^	0.31^***^	0.55^***^	0.60^***^	0.56^***^	0.55^***^	
13 Negative emotions	1.51(0.52)	−0.03	−0.08^**^	−0.06^*^	−0.06^*^	−0.08^**^	−0.33^***^	−0.07^**^	−0.45^***^	−0.45^***^	−0.47^***^	−0.45^***^	−0.38^***^

* *p< 0.05*;

** *p< 0.01*;

*** *p< 0.001*.

### Structural Model Test

M*plus* 8.0 was employed to test the structural model with mediating roles using maximum likelihood estimation. We tested whether the three BPN mediated the relationship between community participation and SWB, while controlling for the roles of covariates in mediators and dependent variables. According to Ryan and Deci (2017), the three basic needs are highly correlated. Thus, the three basic needs were treated as independent mediators; at the same time, the inter-correlations between they were allowed during the model analyzing. According to the M*plus* output, the goodness-of-fit of the structural model of mediation was acceptable: *χ*^2^=526.38, *χ*^2^/*df (68)*=7.74, CFI=0.94, TLI=0.89, RMSEA=0.07 [90% CI: 0.06, 0.07], SRMR=0.04.

### Mediation Effects of BPN

**Figure 2:**
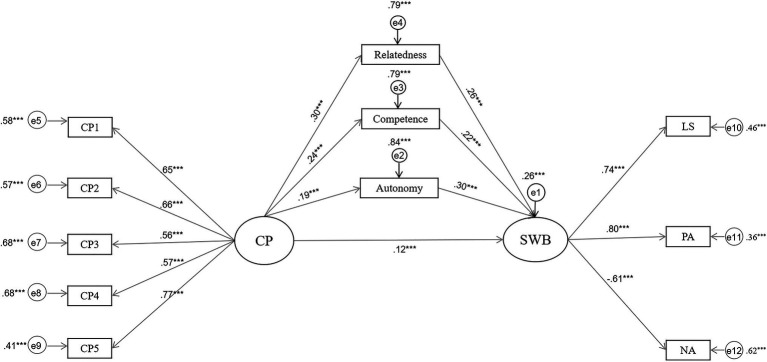
Mediating roles of BPN between CP and SWB. CP, SWP, LS, PA, and NA represent community participation, subjective wellbeing, life satisfaction, positive affect, and negative affect, respectively. ^***^p<0.001.

According to the outcomes of the mediation analysis, the mediating effects of all three BPN were significant, and the ratio of mediating effects in the total effect was 0.18/0.30=0.60. After controlling for the indirect effects of the three BPN, the direct effect of community participation on SWB remained significant. This means that there are other possible mediators to explain the association between community participation and SWB. The results indicated that the 95% bias-corrected confidence interval of the total indirect effect of all three BPN did not contain zero [0.14, 0.22]; nor did those of the indirect effects of needs for autonomy, relatedness, and competence. In addition, the comparison of indirect effects revealed no significant differences in the mediating effects between the three needs (see Tables 4, 5 and Figure 2).

**Table 4:** Total effect, direct effect, and indirect effects of CP on SWB.

	β	*SE*	*t*	*p*	95% CI	
					Lower	Upper
Total effect	0.30	0.03	10.21	0.000	0.24	0.35
Direct effect	0.12	0.02	4.85	0.000	0.07	0.16
**Indirect effects**						
CP→Relatedness→SWB	0.08	0.01	6.16	0.000	0.05	0.10
CP→Competence→SWB	0.05	0.01	5.35	0.000	0.03	0.07
CP→Autonomy→SWB	0.06	0.01	5.46	0.000	0.04	0.08
Total indirect effect	0.18	0.02	8.75	0.000	0.14	0.22
**Contrasts**						
Relatedness Vs. Competence	0.03	0.02	1.65	0.10	−0.01	0.06
Relatedness Vs. Autonomy	0.02	0.02	1.47	0.14	−0.01	0.05
Competence Vs. Autonomy	−0.01	0.01	−0.37	0.71	−0.03	0.02

*CP and SWB represent community participation and subjective wellbeing, respectively*.

**Table 5:** Community participation and subjective wellbeing: Mediated roles of basic psychological needs.

	Relatedness		Competence		Autonomy		SWB		Relatedness		Competence		Autonomy		SWB		SWB	
	β	*t*	β	*t*	β	*t*	β	*t*	β	*t*	β	*t*	β	*t*	β	*t*	β	*t*
Age	0.12	4.33^***^	0.03	0.94	0.11	3.97^***^	0.07	2.30^*^	0.14	5.22^***^	0.04	1.42	0.12	4.49^***^	0.08	2.92^***^	0.01	0.32
Sex	0.20	7.29^***^	0.08	3.07^***^	0.14	4.80^***^	0.08	2.66^**^	0.21	7.79^***^	0.09	3.35^***^	0.14	5.03^***^	0.08	2.89^***^	−0.03	−1.52
Education	0.02	0.75	0.12	4.50^***^	0.03	1.07	0.01	0.19	0.05	1.85	0.15	5.53^***^	0.05	1.74	0.04	1.22	−0.02	−0.97
Material status	0.05	1.96^*^	0.06	2.30^*^	0.06	2.38^*^	0.09	2.84^***^	0.04	1.64	0.05	2.02^*^	0.06	2.19^*^	0.07	2.57^**^	0.04	1.69
Monthly income	0.03	1.17	0.07	2.25^*^	0.09	3.16^***^	0.06	1.93^*^	0.07	2.37^*^	0.09	3.06^***^	0.11	3.85^***^	0.10	3.03^***^	0.03	1.08
Physical condition	0.29	11.33^***^	0.33	13.43^***^	0.30	12.16^***^	0.52	2.42^***^	0.21	7.99^***^	0.27	1.56^***^	0.26	9.66^***^	0.44	16.85^***^	0.25	12.06^***^
CP									0.30	9.91^***^	0.24	7.95^***^	0.19	5.97^***^	0.30	1.21^***^	0.12	4.85^***^
Relatedness																	0.26	7.73^***^
Competence																	0.22	6.84^***^
Autonomy																	0.30	9.95^***^
*R* ^2^	0.13^***^		0.16^***^		0.14^***^		0.31^***^		0.21^***^		0.21^***^		0.17^***^		0.38^***^		0.74^***^	

*CP and SWB represent community participation and subjective wellbeing, respectively*.

* *p< 0.05*;

** *p< 0.01*;

*** *p< 0.001*.

## Discussion

Using a sample of community-dwelling retired residents in China, we examined the level of community participation, the association between community participation and SWB, and the mediating roles of the three basic BPN. The above findings provide a novel perspective for exploring the mechanisms through which community participation affects mental health, as well as insights for policymakers and basic-level governments to improve active aging.

Generally, retirees have a relatively high level of participation in community organization. Especially, retirees tended to participate in volunteer service organizations and organizations based on interests, hobbies, and training. These findings reflect the status quo of participation among retirees in urban areas of China. Traditionally, retirement signified loneliness, isolation, social disengagement, and marginalization from society; moreover, the life of retirees is accompanied by a shrunken social network and a loss of social roles (Cornwell and Waite, 2009). Conventional Chinese notions suggest that older adults gain fulfillment through enjoying a harmonious family life, and caring for their grandchildren often dominates their regular life after retirement. Social participation is mainly limited to irregular or non-organized activities, such as conversing with old friends, visiting relatives, playing cards with neighbors, and so on. In recent decades, this stereotype of what later life should be like has changed. With social development, the implementation of family planning policies, and a weakened concept of familism, older adults have more leisure time and higher motivation to participate regularly in organized activities outside the family. Simultaneously, various types of community organizations have emerged, such as indoor and outdoor activity groups, community service organizations, and unpaid voluntary associations. In China, participation in a variety of organizational activities, especially community activities, has become an integral part of daily life for retirees.

Moreover, community participation was positively associated with three indicators of SWB, independent of socio-demographic and health factors. Hypothesis 1 was confirmed. Older adults with a higher level of community participation reported higher levels of SWB. The basic premises of activity theory and social resource theory offer theoretical support for the findings. By participating in collective or interactive groups, older adults can expand their social resources and offset the losses of social functioning or social networks caused by retirement (Wang, 2016). Participation enlarges the size, scope, and diversity of informal social interactions, which are primary channels for accessing material, social, and information resources (Lin, 2001). The benefits of community participation stem from two basic attributes: functionality and sociality (Park et al., 2013). Functionality meets one’s personal interests. Sociality refers to structural characteristics of community organizations, such as size and diversity, which often satisfy one’s sense of belongingness. It was worth noting that physical health was significantly associated with one’s SWB, which was consistent with previous studies (Pinquart and Sörensen, 2000; Zhang and Zhang, 2015). This finding offers further evidence that physical condition is an important prerequisite for maintaining one’s SWB and mental health (Pinquart and Sörensen, 2000). Therefore, it was reasonable and necessary to control physical health along with other main socio-demographic factors in model analyses.

Three BPN were associated with community participation and SWB, and the mediating roles of all three psychological needs for autonomy, relatedness, and competence were significant. Hypothesis 2, 3, and 4 were confirmed. BPN partially mediated the relationship between community participation and SWB. It is worth noting that BPN do not completely explain the association between CP and SWB, and there are still other potential variables that can be viewed as mediators.

First, it enables retirees to recognize that they can still function in their community and that their life is meaningful and worth living. When individuals feel able to serve their community and help others, their feelings of competence are concomitantly enhanced (Millner et al., 2019). Second, individuals decide whether and in which organizations to participate, which constitutes a self-determined choice to be active in valued community roles, resulting in increased autonomy (Burns-Lynch et al., 2016). Third, community organizations create opportunities to restore retirees’ feelings of competence by presenting them with challenging tasks. For example, the resolution of disputes and conflicts between community residents can enhance retirees’ sense of self-efficacy and sense of ownership. It further helps older adults feel a sense of importance to society, which is closely relevant to fulfilling the needs for competence and autonomy (Wehmeyer et al., 1996; Ohmer, 2007; Piliavin and Siegl, 2007). Finally, community participation provides retirees with opportunities to interact with others and increase their sense of belongingness to the organization and community. Frequent contact between organizational members expands the diversity of an individual’s social network outside the family, and further satisfies their need for relatedness. Participation in community activities gives retirees a sense of community that enhances their feelings of closeness and relatedness to other community members and helps them to avoid rejection and loneliness (Talò et al., 2014). In addition, the test of indirect effects found no significant differences for the mediating effects between the three BPN. This finding is similar to a previous study (e.g., Milyavskaya et al., 2009), in that the effects of the three BPN are consistent in direction and sizes.

### Significances and Limitations

The current study findings have theoretical and practical significance. First, despite considerable research confirming the positive effects of participation on SWB, few empirical studies have examined the mechanisms of this relationship. Our findings reveal this mechanism from the perspective of SDT. That is, the three BPN elucidate why participating in various community organizations positively impacts retirees’ SWB. Engaging in community activities empowers retirees with a sense of control over their own life, enhances self-worth, and provides a sense of connection with neighbors by multiple psychological needs. Social determination theory integrates interpretation mechanisms of multiple theories, such as activity theory, social capital theory, and role theory. Second, this study has important practical implications. Historically, the family unit forms the dominant source of support for older adults in China. However, in recent decades, rapid social changes in China, such as economic progress, urbanization, and longer life expectancy, have led retirees to acquire greater independence in later life. Energetic retirees have more leisure time and a strong motivation to pursue their individual interests and social interactions. Thus, the basic-level governments should fully consider the significance of community-based organizations and the psychological outcomes for retirees when developing policies and measures to promote retirees’ wellness and successful aging. These findings suggest that retirees’ enhanced quality of life requires concerted efforts from government officials, families, and communities.

Several limitations in the design should be mentioned. First, we cannot infer causal relationships between the main variables through a cross-sectional design. The directionality of the pathways between the study variables is unclear. For instance, the thought is also possible only those experiencing psychological need satisfaction is more involved more community participation. Second, in this study, the self-developed community participation questionnaire basically reflected the main aspects of community participation for retirees in China; and psychometric analyses indicated that the questionnaire has good internal consistency and a single-factor structure. However, development of questionnaire did not follow established theories and rigorous procedure of questionnaire development. This slightly rough instrument has limitations in covering all important aspects of community participation of retirees. Thus, a relatively unified general framework and tool to assess the level of community participation is still necessary in future studies. Third, the current study investigated urban retirees in China, who might not be representative of the overall status of the retired population in China. Future research should be conducted among a more representative population through a strict sampling. Furthermore, given prevalence of COVID-19, researchers can only adopt the mode of online survey with smartphone to collect data; however, the usage of smartphone among older people is not as widespread as among younger people (so called digital divide), which limits the generalization of findings. In addition, the effect of community participation on SWB and relevant mechanisms may differ across cultures. For example, residents in a country with a collectivist culture are more likely to participate in volunteer activities than those in a country with an individualistic culture (Parboteeah et al., 2004). People in individualistic societies value self-reliance and independence in their social lives, and thus have greater needs for autonomy. However, people in collectivist societies value interdependence and team spirit, and therefore have higher needs for relatedness. Future studies should consider cultural differences and investigate these concepts.

### Conclusion

This study sought to elucidate the significance of participating in community organizations, its effects on SWB, and the mediating mechanisms thereof among urban retirees in China. Despite the above limitations, our study achieved several goals. First, we gained insight into the basic status of retirees’ participation in community affairs and activities in urban China. Second, our study supported the positive effects of community participation on SWB. Third, and most importantly, we found that BPN mediated the relationship between community participation and SWB. Community participation benefits the mental health of retirees by their needs for autonomy, competence, and relatedness. With age and the subsequent decline in physical function, older adults may be more affected by community or neighborhood environments. They are also more likely to rely on their residential community or adjacent neighborhoods as a source of social support and activity than young adults (Glass and Balfour, 2003). Community organizations as social environmental factors fulfill multiple individual needs, which is promising for improving the quality of life of retirees.

## Data Availability Statement

The original contributions presented in the study are included in the article/supplementary material, and further inquiries can be directed to the corresponding author.

## Ethics Statement

The studies involving human participants were reviewed and approved by the ethics committee of Institute of Psychology, Chinese Academy of Sciences. The patients/participants provided their written informed consent to participate in this study.

## Author Contributions

CL performed the measurement, conducted the analyses, and drafted the manuscript. ZZ conceived the study, conducted the analyses, and drafted the manuscript. Both authors read, modified, and approved the final manuscript.

## Funding

This work was supported by the grants of the National Natural Science Foundation of China (grant numbers 71774157 and 71273255) and the Independent Deployment Foundation of Institute of Psychology, Chinese Academy of Sciences [grant number E0CX083008].

## Conflict of Interest

The authors declare that the research was conducted in the absence of any commercial or financial relationships that could be construed as a potential conflict of interest.

## Publisher’s Note

All claims expressed in this article are solely those of the authors and do not necessarily represent those of their affiliated organizations, or those of the publisher, the editors and the reviewers. Any product that may be evaluated in this article, or claim that may be made by its manufacturer, is not guaranteed or endorsed by the publisher.
